# A systematic search for new mammalian noncoding RNAs indicates little conserved intergenic transcription

**DOI:** 10.1186/1471-2164-6-104

**Published:** 2005-08-05

**Authors:** Tomas Babak, Benjamin J Blencowe, Timothy R Hughes

**Affiliations:** 1 Banting and Best Department of Medical Research, 112 College St., Toronto, ON M5G 1L6 Canada; 2 Department of Medical Genetics and Microbiology, 10 King's College Circle, Toronto, ON M1R 4F9 Canada

## Abstract

**Background:**

Systematic identification and functional characterization of novel types of noncoding (nc)RNA in genomes is more difficult than it is for protein coding mRNAs, since ncRNAs typically do not possess sequence features such as splicing or translation signals, or long open reading frames. Recent "tiling" microarray studies have reported that a surprisingly larger proportion of mammalian genomes is transcribed than was previously anticipated. However, these non-genic transcripts often appear to be low in abundance, and their functional significance is not known.

**Results:**

To systematically search for functional ncRNAs, we designed microarrays to detect 3,478 intergenic and intronic sequences that are conserved between the human, mouse, and rat genomes, and that score highly by other criteria that characterize ncRNAs. We probed these arrays with total RNA isolated from 16 wild-type mouse tissues. Among 55 candidates for highly-expressed novel ncRNAs tested by northern blotting, eight were confirmed as small, highly-and ubiquitously-expressed RNAs in mouse. Of the eight, five were also detected in rat tissues, but none were detected at appreciable levels in human tissues or cultured cells.

**Conclusion:**

Since the sequence and expression of most known coding transcripts and functional ncRNAs is conserved between human and mouse, the lack of northern-detectable expression in human cells and tissues of the novel mouse and rat ncRNAs that we identified suggests that they are not functional or possibly have rodent-specific functions. Our results confirm that relatively little of the intergenic sequence conserved between human, mouse and rat is transcribed at high levels in mammalian tissues, possibly suggesting a limited role for transcribed intergenic and intronic sequences as independent functional elements.

## Background

Comparative genomics has revealed that approximately 5% of the mammalian genome is under purifying selection [1,2]. While exons make up roughly 1.5% of the genome [3], relatively little is known about the role of the remaining 3.5% of the highly conserved genomic regions, and even less about the functional potential of evolutionarily-diverged intergenic sequences. Large-scale microarray tiling analyses (i.e. using a set of probes designed to detect all or most of a targeted genome or genomic region), as well as high-throughput cDNA sequencing efforts, have indicated that the "transcriptome" is significantly larger than was previously appreciated, although the functional significance of the vast majority of the novel, apparently noncoding (nc) transcripts detected by these approaches has remained elusive [4-8]. To date, several studies have reported large-scale tiling efforts of the human genome [4,5,9,10]. In all cases a significantly higher proportion of transcribed sequence was reported than could be accounted for by existing exon annotation data, and much of the remainder did not appear to encode protein [4]. Comparison of datasets suggests that a high proportion of the novel transcripts are specific to tissues or cell lines [4,9]. This trend was particularly evident for cell lines, where novel cell-line specific transcripts were even more numerous than annotated cell-line specific exons [4], implying that many of these transcripts may not have endogenous functions in whole organisms. Further supporting this possibility was the observation that the majority of the novel transcripts were detected at very low levels [5].

A second source of evidence for a more extensive transcriptome arises from large-scale cDNA compilation efforts. The mouse cDNA sequencing effort led by the RIKEN Consortium identified 60,770 unique cDNA transcripts from a variety of mouse tissues and cell lines [7]. Approximately half (33,409 sequences) were derived from unique genomic locations (Transcriptional Units), of which 15,815 did not map to known or predicted coding genes in mouse [7]. Further refinement identified a set of 4,280 mRNA-like noncoding RNAs which had no homology to any known protein sequences and comprised of sequences mapped to regions located between predicted exon boundaries [6]. Many of these sequences were reported elsewhere in EST databases and displayed features of polymerase II transcripts [6]. However, unlike protein-coding mouse genes, of which 99% have homologs in the human genome [1], only 10.6% of the 4,280 apparent nc transcripts were represented by homologous sequences in the human genome [6,7]. In fact, Wang et al. [11] demonstrated that most of these transcripts are no more conserved than intergenic sequence in general, and less conserved than a comprehensive set of 321 known ncRNAs with established functional roles. In addition, expression profiling of a different but overlapping (FANTOM1) subset of cDNAs that do not map to known ESTs or protein sequences (3,388), revealed that most transcripts in this uncharacterized class were present at low abundance [12]. Collectively, these results demonstrate the transcription of uncharacterized sequence, but raise questions about the functional relevance of the novel "noncoding" set.

One possible explanation for the observed low-level expression of a much larger fraction of genomes than can be accounted for by known genes comes from the recent discovery of a nuclear posttranscriptional quality-control pathway that degrades "cryptic unstable transcripts" (CUTs) in yeast [13]. CUTs are transcribed by Pol II and are detectable by both microarrays and RT-PCR in wild-type yeast, and also appear to be frequently represented as single tags in SAGE libraries, but are undetectable by Northern blotting and do not contain significant open reading frames. However, in mutants in the quality-control pathway, they appear as a smear on Northern blots due to the fact that they have heterogeneous 3' ends [13]. The fact that a posttranscriptional quality control exists to prevent accumulation of CUTs suggests that they are aberrant and predicts that there should be little selection pressure on their expression. Moreover, these observations suggest that nonfunctional transcripts might be distinguished from *bona fide *functional transcripts on the basis of formation of a discrete species on Northern blotting, and by conservation of expression among different organisms.

In this study, we describe a systematic approach to predict and screen novel ncRNA transcripts in the mouse genome. We first identified non-exonic sequences that are most likely to encode functional ncRNAs (functional transcripts that do not encode proteins) by using the program QRNA, which searches for conserved regions with compensatory mutation patterns that are consistent with the evolutionary conservation of secondary structure in functional noncoding sequences [14]. These are hallmarks of most known functional ncRNAs, and QRNA has been used successfully to identify novel structural ncRNAs in *E. coli *[15] and *S. cerevisiae *[16]. However, even in these organisms, which have relatively compact genomes, a high false-positive prediction rate was observed [16], which presents a challenge for screening large genomes. We therefore used a custom oligonucleotide microarray [17] as an initial high-throughput screen for expression. We then tested the 55 highest-expressed candidates to ask whether they are detectable as discrete species on Northern blots. We report eight novel mouse transcripts identified using this approach. However, none of the eight appears to be expressed in humans, casting doubt on their role as independent functional elements. Taken together with the low proportion of intergenic sequences that we detected, our results suggest that much of the recently-discovered expanded transcriptome [4-7,9,10] may correspond to cryptic transcripts [13], suggesting a limited role for transcribed intergenic and intronic sequences as independent functional elements.

## Results

### Predicting novel ncRNA candidates

**Figure 1:**
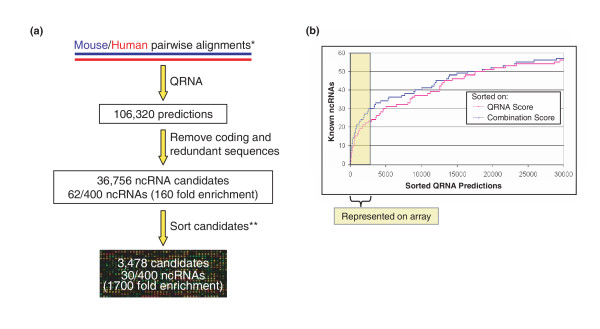
Summary of computational prediction and sorting of novel mammalian noncoding RNA. **(a) **Whole genome alignments were downloaded from UCSC [18], subset to regions of greater than 85% sequence identity, and analyzed with QRNA [14]. Removal of redundant and coding sequences left 36,756 ncRNA candidates containing 62 known ncRNAs (of approximately 400 known ncRNAs). Candidates were then sorted and the top 3,478 predicted RNAs, which contained 38 known ncRNAs (representing a 1,700 fold enrichment of real ncRNAs over random selection from genomic sequence), were selected for further screening. **(b) **Sorting was based on the QRNA score, stability (measured as a predicted free energy change using Mfold [44]), overlap with rat-mouse QRNA predictions, and genomic clustering (many predictions close to one another are likely the same transcript). This combination of four criteria was more powerful in identifying real ncRNAs than using the QRNA score alone. *UCSC [18], **See **(b)**.

We identified novel structural ncRNA candidates on the basis of two features: high sequence conservation and a mutation pattern consistent with sequences being under selective pressure to maintain a conserved secondary structure [see Methods for details]. Figure 1 outlines the computational screen we employed for finding novel ncRNAs. We obtained human-mouse pairwise sequence alignments from UCSC [18] and subset them to alignments with a minimum of 85% sequence identity using a 200 nt scanning window. This eliminated sequences that are unlikely to be under evolutionary selection [1,11] and reduced the dataset to a computationally manageable size. We used QRNA v.1.1 [14-16] to screen the alignments for putative ncRNAs. This generated 106,320 predicted ncRNAs. We then removed redundant sequences and predicted ncRNAs with sequences similar or identical to coding genes annotated in the Mouse RefSeq mRNA database [19], RIKEN cDNA [7], Mouse Protein NR database [20], or coding genes in other organisms annotated in GenBank [21]. The remaining 36,756 predicted ncRNAs were sorted by logistic regression using four parameters that we identified to be useful for distinguishing known ncRNAs from QRNA predictions of putative new ncRNAs: 1) the QRNA logodds RNA score; 2) the thermodynamic stability of the predicted secondary structure of each prediction; 3) a genomic clustering score of closely mapped predictions on the genome (presumed to be multiple regions of a longer ncRNA transcript); and 4) an overlap between mouse-human and mouse-rat QRNA predictions (processed similarly to mouse-human alignments). The QRNA score was the strongest indicator of defined ncRNAs (data not shown), but combining the additional parameters increased the sorting power, especially for the top 10% of the predicted RNAs (Fig. 1B). Other parameters such as GC content and length of the sequences did not improve the sorting (data not shown). To further characterize the set of predicted ncRNAs, we screened them computationally for tRNAs and snoRNAs [22,23] and searched for similar sequences in the RIKEN FANTOM2 mRNA-like ncRNA collection. For a summary of these features, see Additional file 1.

### Array design

Due to the generic nature of the algorithm, QRNA has a high false positive rate, much higher than coding gene-finding algorithms, thus experimental validation is essential. Using the prediction scheme and sorting criteria described above, we designed a microarray to detect 3,478 QRNA predictions with properties most indicative of ncRNAs. The microarray contained probes for the top 9.5% of the total ncRNA predictions, and probes for 38 known ncRNAs [see Additional file 2]. The design included 20,867 complementary DNA probes to the QRNA predictions (with six probes per prediction; three for each orientation), 200 random probe sequences and 305 intergenic probes that served as negative controls, and 705 positive control probes tiled across U4, U5 and mature rRNA transcripts. A list of all microarray probes is included in Additional file 3.

### Analysis of RNA from diverse mouse tissues

**Figure 2:**
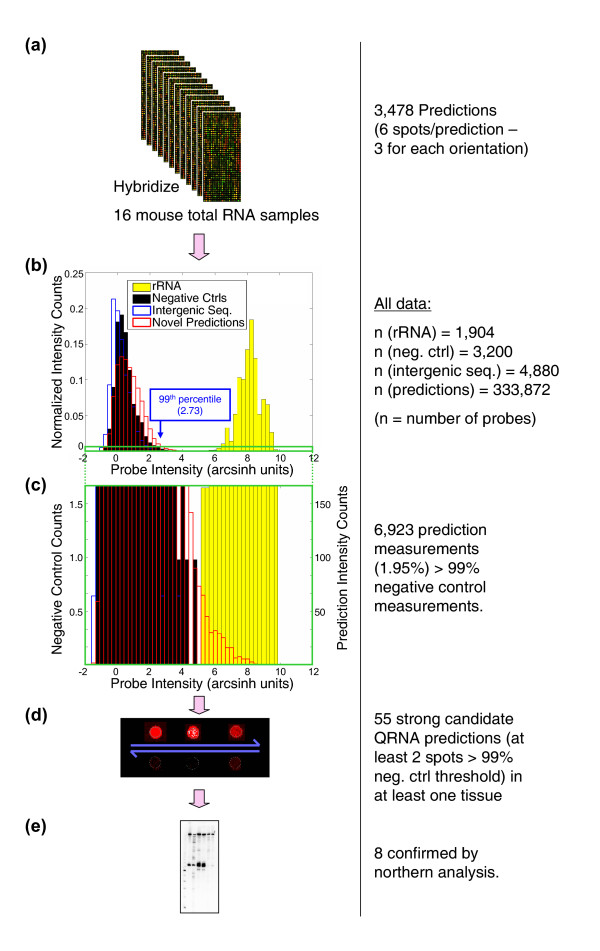
Screening, selection, and confirmation of novel ncRNA predictions. **(a) **Total RNA from 16 tissues was hybridized to custom Agilent microarrays containing probes to the QRNA predictions. **(b) **Most intensity measurements overlapped negative-control intensities (both random probe sequences and probes corresponding to randomly sampled intergenic regions), although a right-tailed distribution overlapping rRNA levels indicated detection of potential abundant novel transcripts. **(c) **Expanded y-axis region from **(b)**, axes denote absolute probe intensity counts. **(d) **Sample schematic of microarray spots corresponding to a transcript that was tested further by northern analysis. **(e) **55 transcripts in total were screened by Northern analysis.

Since ncRNAs can be expressed in a tissue-or developmental-stage-specific manner [24-29] we screened 16 mouse tissues/organs encompassing a variety of tissue sources, including two embryonic stages of development (Table 1). The intensity distribution over all measurements is shown in Figure 2. As expected, the majority of QRNA-prediction intensity measurements overlapped with the negative control probe measurements, presumably due to the high false-positive rate inherent in generating the predictions. However, the distribution was skewed to the right tail of the plot (i.e. higher intensity), and twice as many of measurements from the predicted ncRNAs were above the 99% negative control threshold (generated using random sequence probes) than would be expected based on a random distribution (Fig. 2). Of the 38 known ncRNAs that were among the QRNA predicted RNAs [see Additional file 2], we detected 15, including several snoRNAs, tRNAs, a Hox antisense transcript, and miRNAs, using the same intensity cutoff used for selecting novel candidates (see below). This illustrates that the sensitivity of this technique is sufficient to detect most known ncRNA types. We have also used this technique to survey miRNA expression [24].

**Table 1:** List of tissues used in microarray expression analysis

Tissues/Organs/Cells screened for novel noncoding RNAs
Bladder
Brain
Embryonic Stem Cells
Femur
Heart
Intestine
Liver
Lung
Mammary Gland
Muscle
Stomach
Teeth
Testis
12.5-day Embryo
15-day Embryo
9.5-day Placenta

### Validation by northern blotting

**Figure 3:**
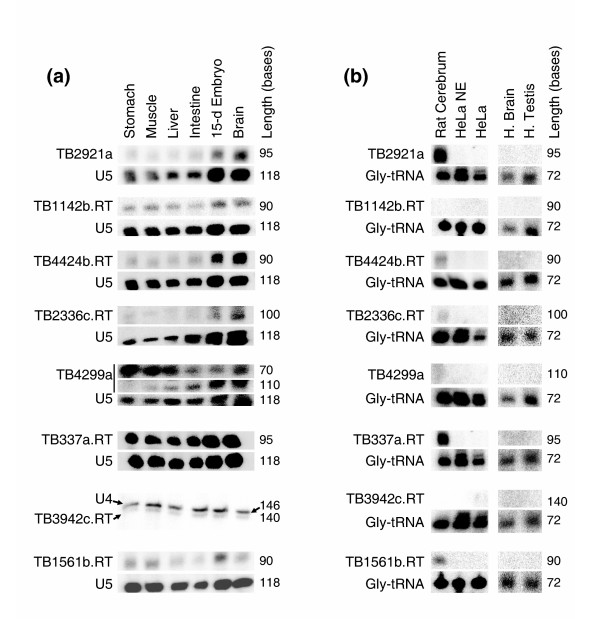
Abundantly and ubiquitously expressed novel mouse transcripts are not expressed in human tissues or cells. **(a) **Northern analysis of mouse transcripts using mouse-specific probes. U5 and U4 probes were used as loading controls as indicated and were co-hybridized with test probes. **(b) **No signal was detected in Northern analyses using human-specific probes. Human tissues were analyzed with a longer exposure (right panel) since short RNAs were slightly underrepresented in these commercially-obtained samples. Mouse-, and human-specific probe sequences complementary to the novel ncRNA predictions and images of all full-scale northern blots are available in Additional files 4 and 5.

Although our microarray data indicated that many of the measurements arise from real transcripts, noise (e.g. spurious cross-hybridization) could also account for some proportion of the high-intensity measurements. Furthermore, microarray results cannot differentiate between a single RNA species and a heterogeneous population. We therefore used Northern blotting to validate our candidate novel ncRNAs. Northern blotting is more quantitative than RT-PCR, since there is no exponential amplification step. It is less sensitive for the same reason; however, using our methods, we have been able to detect all types of transcripts including structural ncRNAs, miRNAs, and mRNAs ([24,30] and data not shown). Importantly, since Northern blotting reveals the size of the RNA species detected, it can distinguish whether there is a single RNA product species and a heterogeneous transcript population. We tested all predicted ncRNAs detected by at least two of three probes (all in the same orientation) displaying signals above the 99%-negative control intensity threshold in at least one tissue. In total, this included 55 novel predicted ncRNAs, of which most appeared to be ubiquitously expressed. Northern analysis on this subset confirmed 8 novel transcripts (Fig. 3), all of which were detected ubiquitously in total RNA isolated from 16 wild-type mouse tissues. All eight transcripts were between 70 and 140 nt in length, none had tRNA or snoRNA structural or sequence characteristics, and five were located in intronic regions. It is possible that additional RNAs are expressed at low levels that are detectable by microarray and/or RT-PCR but not by Northern blotting, especially if they are heterogeneous in length [13]. We did not pursue this possibility, since it seemed that transcripts undetectable by Northern blotting are less likely to represent bona fide ncRNAs.

### Expression of novel mouse transcripts is not conserved in human cells/tissues

Nearly all sequence-conserved coding genes between human and mouse have a conserved expression pattern across multiple tissues [31]. Although they have not to our knowledge been comprehensively analyzed, ncRNAs are also generally expressed similarly across related species [32] and since most are required for cell proliferation, they tend to be expressed in all tissues, as were all eight of the novel transcripts we observed. However, Northern blotting revealed that none of the eight novel mouse transcripts were expressed at detectable levels in HeLa cells or in human tissues (Fig. 3). Moreover, only five of these were detected in rat (Fig. 3). Images of all full-scale Northern blots shown in Figure 3 and other supporting Northern data is available in the supplementary data [see Additional file 4].

**Figure 4:**
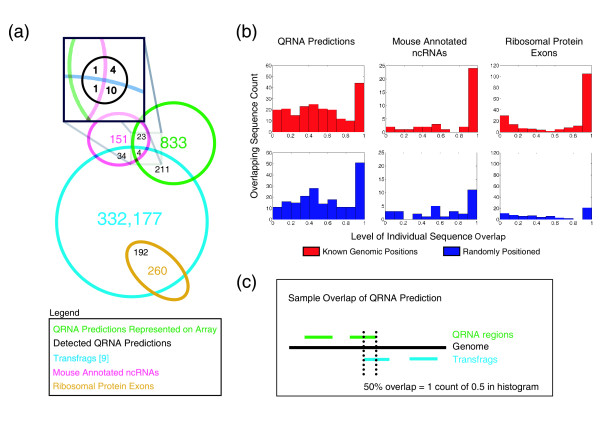
Overlap between known and predicted ncRNA types. **(a) **3,478 QRNA predictions, 390 mouse annotated ncRNAs, and 716 human ribosomal exons were mapped to the human genome (hg17) regions surveyed by Cheng et al. [9] using poly-A minus RNA derived from HepG2 cells. 833 QRNA predictions, 151 mouse ncRNAs, and 260 exons are located in regions surveyed by Cheng et al. **(b) **Shown are distributions of the percentage of overlap of each sequence with a transfrag for actual genomic positions versus randomly positioned sequences in regions surveyed by Cheng et al. [9]. **(c) **Schematic of how an overlap was calculated for one QRNA prediction that overlaps a transfrag. This was repeated for all overlaps and the distributions are shown in **(b)**.

We also compared our 3,478 QRNA predictions and the RNAs we verified with recently published high-density human tiling data from Cheng et al. [9]. We subset our QRNA predictions to regions surveyed by Cheng et al. [9] (i.e. the non-repetitive regions of human chromosomes 6, 7, 13, 14, 19, 20, 21, 22, X, and Y). We considered the "transfrags" (i.e. transcribed fragments: any transcribed genomic region) described by Cheng et al. [9] in poly-A-minus RNA from HepG2 cells, which should be most comparable to our data (Fig. 4A). We confirmed that the Cheng et al. [9] "transfrags" encompassed a larger number of known noncoding RNAs and mRNA exons than was obtained from random positioning of sequences of the same length (Fig. 4B), although the vast majority of "transfrags" do not overlap any annotated features. We did not see a marked difference in the overlap between the Cheng et al. "transfrags" and our QRNA predictions (Fig. 4B). This indicates that the "transfrags" are not enriched for conserved sequence with conserved secondary structure, consistent with our data showing a lack of conserved expression of our northern-confirmed QRNA transcripts in human tissues and cells. Only one of the eight northern-confirmed novel mouse transcripts we verified mapped to the regions surveyed by Cheng et al. [9] and it did not overlap a "transfrag", also consistent with our results (Fig. 3).

## Discussion

Using comparative genomics and an established ncRNA search method modified for high-throughput screening, we report eight novel mouse ncRNA transcripts that are all relatively short, ubiquitously expressed, and abundant. Despite their sequence and secondary structure conservation, none of the transcripts were expressed at detectable levels in human cells and tissues.

Given the large search space incorporated in this analysis, our results indicate that little intergenic or intronic sequence is expressed as distinct, stable transcripts at levels comparable to the expression of most known functional RNAs. This deduction contrasts somewhat with the conclusions of recent studies employing tiling arrays or large-scale cDNA sequencing [5,7,9,10]. Because results from other studies were obtained and validated in different ways, we cannot confirm or refute the basic observations of any other study. Numerous explanations exist for the breadth of the emerging transcriptome [10]. Nonetheless, we propose that claims regarding a dramatically larger transcriptome than is accounted for by current annotations should be addressed with scrutiny, particularly with regard to functional potential. Several factors indicate that a significant proportion of the newly measured transcripts may either be spurious or non-functional: 1) transcriptionally active regions identified in tiling analyses and potentially noncoding cDNAs are generally detected in low abundance [4,6]; 2) in yeast, many intergenic regions are also transcribed at low levels, apparently as heterogeneous species, and there is a specific mechanism for degrading these transcripts [13]; 3) much of the mammalian data available is from cell lines, including a high proportion of tumor-derived cell lines [5,9], which may lack the same degree of quality-control as found in normal cells and tissues; 4) most are relatively short (i.e. sequenced transcripts are shorter than the average coding gene [7], as are transcripts identified from tiling, which are on average less than 200 nt [4]); 5) potentially noncoding cDNAs correspond to regions not conserved at the sequence level [6,7]) and have evolved at a non-selective rate [11]; 6) there is little evidence for cross-species expression (only 2.6% of noncoding mouse cDNAs can be mapped to human ESTs [6,7]); 7) 70% of intergenic "transfrags" corresponding to novel transcribed regions could not be detected by northern analysis [4]); 8) the "transfrags" do not appear to be enriched in sequences with conserved secondary structures (Fig. 4), which is a hallmark feature of known structural ncRNAs [33].

How can we distinguish *bona fide *functional transcripts, in the absence of directed genetic experimentation? Sequence conservation alone is apparently not sufficient to distinguish sequences with critical functions, as large-scale non-genic deletions encompassing highly conserved regions can be tolerated in mice without detectable fitness disadvantages [34]. The presence of conserved expression improves the likelihood that sequence-conserved regions are functional since most characterized RNA classes, including coding mRNA, and noncoding rRNA, tRNA, snRNA, snoRNA, and miRNA, generally exhibit conserved expression patterns across evolutionarily-related species [31,32,35,36]. Of the few characterized mRNA-like ncRNAs, some also have conserved expression patterns [29,32,37]. The lack of conserved expression of the eight transcripts identified in our study, despite a high sequence and structural similarity, suggests that they are not functional, although it is possible that a subset of functional transcripts have species-or lineage-specific functions despite their high degree of sequence conservation. For example, a subset of the ncRNAs detected in mouse in our study were also detected in rat tissues, suggesting the possibility of conserved functions restricted to the rodent lineage. However, in reported cases of mouse-specific ncRNAs, such as BC1, Tsix, CIOR, and t-ncb [32], the RNAs were not conserved at the sequence level which is likely the reason for mouse-specific ncRNA differential regulation.

More cross-species and non-biased expression data is required to definitively address the likelihood of functionality of emerging transcriptomes. The most comprehensive approach will likely be an extension of whole genome tiling microarray analyses [38] using RNA derived from endogenous tissues from a variety of organisms. The approach of hybridizing covalently labeled total RNA (applied in this paper), as opposed to cRNA or cDNA derived from poly-adenylated RNA, presents a potential improvement to the unbiased nature of tiling analyses, since there is no amplification bias and strand information is retained. An added dimension of conserved expression will enable focused functional experimentation on transcripts that are likely to be important, although our data indicates that these cases will be the exception rather than the rule.

## Conclusion

With the application of high-throughput transcriptional analyses it has been reported that more sequence is transcribed than was previously appreciated, with some estimates exceeding twice that of currently annotated transcripts. In a systematic search for sequence-conserved transcripts with hallmarks of structural ncRNAs, we identified only eight novel mouse transcripts with ubiquitous and abundant expression. This indicates that very little intergenic sequence is transcribed at high levels. Furthermore, despite meeting the stringent requirements of characterized ncRNAs, none of these eight transcripts were expressed at detectable levels in human cells or tissues. This suggests that these transcripts are unlikely to have conserved functional roles. We propose that newly-identified transcriptomes should be viewed with scrutiny, particularly with regard to function, until it is determined that they are functional or at least display properties of known functional elements.

## Methods

### Predicting novel ncRNAs

Whole genome pairwise human-mouse alignments were downloaded from the UCSC Genome Bioinformatics website ([18]; build 32, Nov. 2003). Repeat-masked [39] alignments were subset to segments with a minimum of 85% sequence identity. QRNA v.1.1 [14] was used to score the alignments for noncoding RNA potential using settings determined to work optimally on a test set of ncRNAs embedded in random alignments of equivalent sequence identity (default settings with -w 100 -x 50). The processing time was approximately 14 days on a 20-processor (1 GHz) linux cluster. Overlapping sequences with a positive logodds RNA score were concatenated into one sequence which was assigned the highest score of the original component sequences.

### Selection of candidate ncRNAs

QRNA predictions were filtered by alignment to a variety of coding sequence databases using BLAT [40] with a default score cutoff of 30 ([alignment length] – [number of mismatches]) and a minimum sequence identity of 60% (-minIdentity = 60). The databases included: Mouse RefSeq mRNA [19] (build 29), ENSEMBL coding transcripts [41], RIKEN cDNA [7], ESTs [42] (download date: Nov. 2003), Unigene [43] (Nov. 2003), Protein NR [20] (build 29), and Genbank NT Database [21] (Nov. 2003). Redundant QRNA predictions and predictions that aligned to annotated coding sequences were removed. The remaining set was screened for tRNAs and Box C/D snoRNAs using tRNAScan SE [22] and snoscan [23] respectively. Sequences were extended by 100 bases in both directions from the genome to ensure complete coverage of potential tRNAs or snoRNAs.

QRNA predictions that did not map to annotated coding genes were sorted on a combination of criteria to maximize selection of known ncRNAs (as compiled in [24]). Sorting parameters included the minimum free energy as predicted by Mfold [44], overlap between mouse-human and mouse-rat QRNA predictions (blast, e-threshold 10^-4^), and proximity to adjacent predictions in the genome. Genomic proximity was scored by adding the number of QRNA predictions within 1000 bp of each other in the genome. Mouse-rat alignments [18] were processed identically to mouse-human alignments. Multiple linear regression was used to assign weights to these parameters in addition to the QRNA logodds RNA score and were subsequently used to calculate an overall score for each QRNA prediction. The top 3,478 predicted RNAs (limited by space on the array) were analyzed further by microarray. These contained 38 known ncRNAs of the approximately 400 known ncRNAs, representing a 1,700-fold enrichment. The level of enrichment was calculated as the ratio of the proportion of nucleotides that are real ncRNAs in the QRNA predicted set to the proportion of nucleotides of all known ncRNAs of the mouse genome (i.e. how much more likely one could select a nucleotide belonging to a known ncRNA in the QRNA set over the whole genome).

### Microarray design

Six probe sequences were allotted for each ncRNA prediction; three for each orientation. Complementary DNA probes were designed to maximize spatial coverage of each predicted sequence and were normalized by length (i.e. probe lengths were adjusted) to a uniform melting temperature of 60°C. Probe sequences were on average 26.9 nt and were concatenated to 60 nucleotides. Probe sequences were submitted to Agilent Technologies for microarray production (Palo Alto, California). The designs included 200 60-mer probes containing random sequences, which were used as negative controls, and 696 positive control probes tiled across U4 and U5 snRNAs and 18S and 28S rRNAs. Additional file 3 contains a list of all of the probe sequences.

### RNA extraction, labeling, and hybridizations

HeLa nuclear extract (NE) was prepared as described previously [45]. Total RNA from HeLa cells, HeLa NE, and mouse tissues was extracted using Trizol (Invitrogen) according to the manufacturer's instructions and was treated with DNase I (Fermentas). Total RNA derived from human tissues was purchased from Clontech (BD Biosciences, Mississauga, ON) and Ambion (Austin, TX). Integrity of rRNA was confirmed on 1% agarose-formaldehyde gels. 7 μg of total RNA was chemically labeled with Ulysis Alexa Fluor 546 or Ulysis Alexa Fluor 647 (Ulysis) according to manufacturer's instructions. This protocol labels G residues [46], and there were no predicted RNAs that lacked G residues. Samples were resuspended in 0.5 mL of hybridization buffer (1 M NaCl, 0.5% sodium sarcosine, 50 mM N-morpholino ethane sulfonate, pH 6.5, 33% formamide and 40 μg salmon sperm DNA), denatured by heating at 65°C for 5 minutes, and snap-cooled on ice prior to hybridization. Hybridizations were carried out for 16–24 h at 42°C in a rotating hyb oven. Slides were then washed (rocking ~30 seconds in 6× SSPE, 0.005% sarcosine, then rocking ~30 seconds in 0.06× SSPE) and scanned with a 4000A microarray scanner (Axon Instruments, Union City, CA).

### Microarray data processing and normalization

TIFF images were quantified with GenePix 3.0 (Axon Instruments, Union City, CA). Individual channels were spatially detrended (i.e. overall correlations between spot intensity and position on the slide removed) by high-pass filtering [47] using 5% outliers. The 16 individual channels were then normalized using Variance Stabilization [48,49] and transformed to arcsinh values, which are similar to natural log values but are tolerant of negative numbers emerging from high-pass filtering.

### Northern blotting

7 μg of total RNA from each tissue was separated on 10% polyacrylamide/TBE/urea gels, and electroblotted to Hybond N^+ ^or Hybond-XL membranes (Amersham) using asemi-dry transfer apparatus(Bio-Rad) in 0.5X TBE according to the manufacturer's instructions. The membranes were UV cross-linked using a Stratalinker (Stratagene), hybridized overnight at 42°C in Church buffer with 5'-^32^P-end-labeled oligonucleotide probes, and washed with 2X SSC, 0.1% SDS and 0.1X SSC, 0.1% SDS for 5 minutes each at 42°C. Results were analyzed using a Phosphorimager (Bio-Rad Personal FX). Oligonucleotide probe sequences are listed in Additional file 5.

### Calculating overlap with human tiling analyses

The 3,478 QRNA predictions analyzed by microarray were mapped to the mouse-human UCSC genomic alignments (mm6-hg17) and were subset to the same regions analyzed by Cheng *et al*. [9] (i.e. not repetitive regions, for example), which were determined from the probe positions used in the tiling analysis (coordinates were converted to the hg17 genome release using the UCSC LiftOver tool [17]). The tiling dataset we focused on was generated using nuclear poly-A minus RNA derived from HepG2 cells. For QRNA predictions that overlapped with a transfrag, the degree of overlap was calculated as a percentage of the length of the QRNA prediction that overlaps the transfrag. The distribution of QRNA overlaps was compared to overlaps from randomly positioned QRNA predictions in the human-surveyed regions. The random set consisted of a set of sequences identical in length to the actual QRNA predictions, but with randomized positions in the human surveyed regions. The same analysis was repeated using 390 (151 mapped to human surveyed regions) mouse annotated miRNAs, snoRNAs, snRNAs, and tRNAs downloaded from NONCODE [47] and Rfam [48] databases, and 716 human ribosomal protein exons (260 mapped to human surveyed regions) annotated in the Refseq database [18].

### Data availability

All supplementary data is available at . The microarray design has been submitted to NCBI GEO in MIAME format under accession GSE2366. The 8 novel transcripts have been submitted to GenBank [Genbank:AY954743 – Genbank:AY954751].

## Authors' contributions

TB carried out the data compilation, microarray analysis, and data analysis. BB and TH coordinated the study. All authors contributed to preparation of the manuscript.

## Supplementary Material

Additional File 1 Summary of array-tested ncRNA predictions. Click here for file

Additional File 2 List of known mouse ncRNAs represented on array. Click here for file

Additional File 3 Microarray probe sequences. Click here for file

Additional File 4 Whole-blot northern data. Click here for file

Additional File 5 List of northern probe sequences.
Click here for file
