# Supplementary material for: A systematic search for new mammalian noncoding RNAs indicates little conserved intergenic transcription
Source: BMC Genomics. 2005 Aug 5;6:104. doi: 10.1186/1471-2164-6-104 (PMC1199595; doi:10.1186/1471-2164-6-104)
Supplement: Additional File 4 — Whole-blot northern data. [file 1471-2164-6-104-S4.pdf]

337a.RT

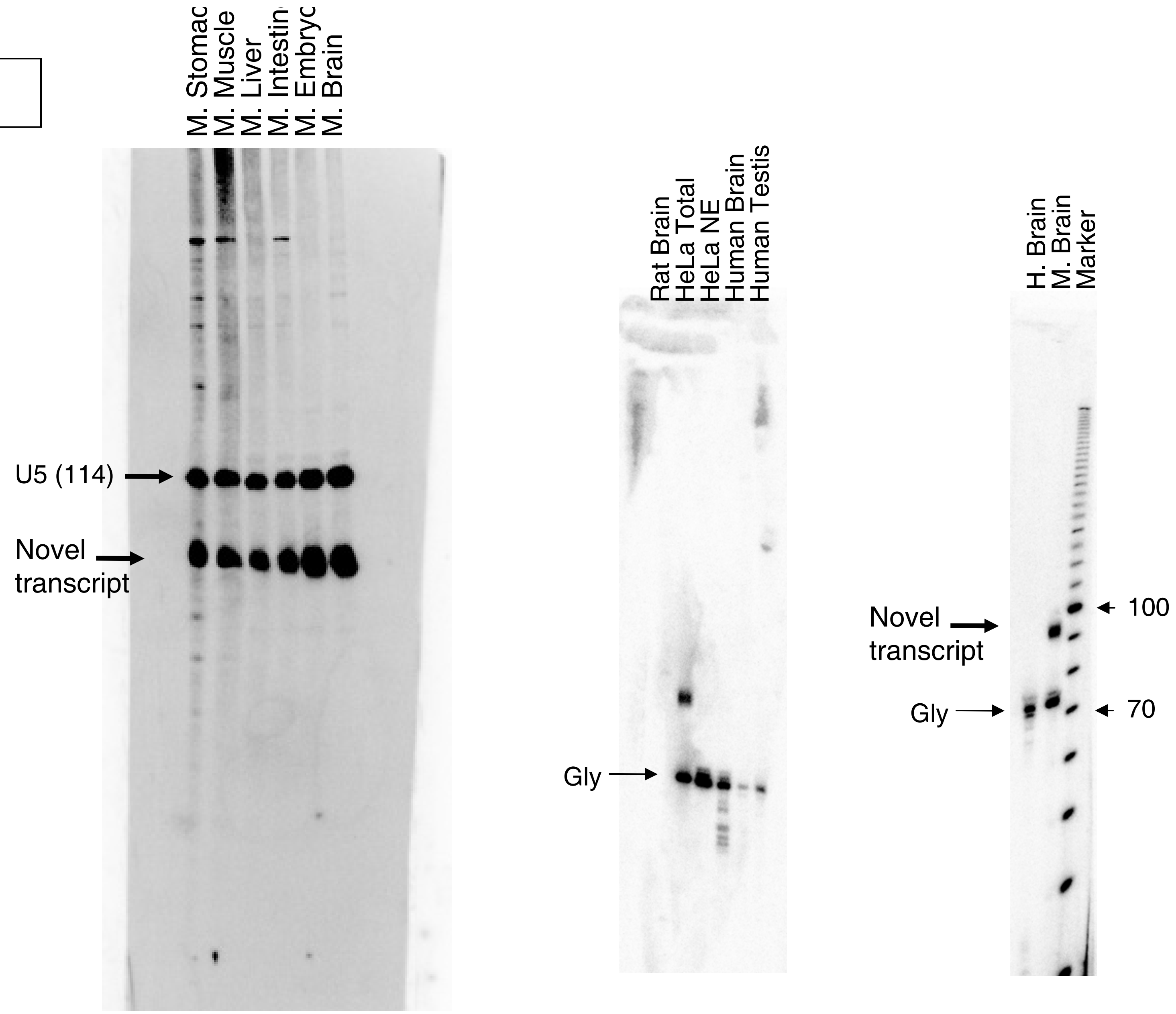

Hybridized with:	Mouse 337a.RT U5	human 337a.RT<br>Gly-tRNA	Mouse 337a.RT,<br>human 337a.RT<br>Gly-tRNA

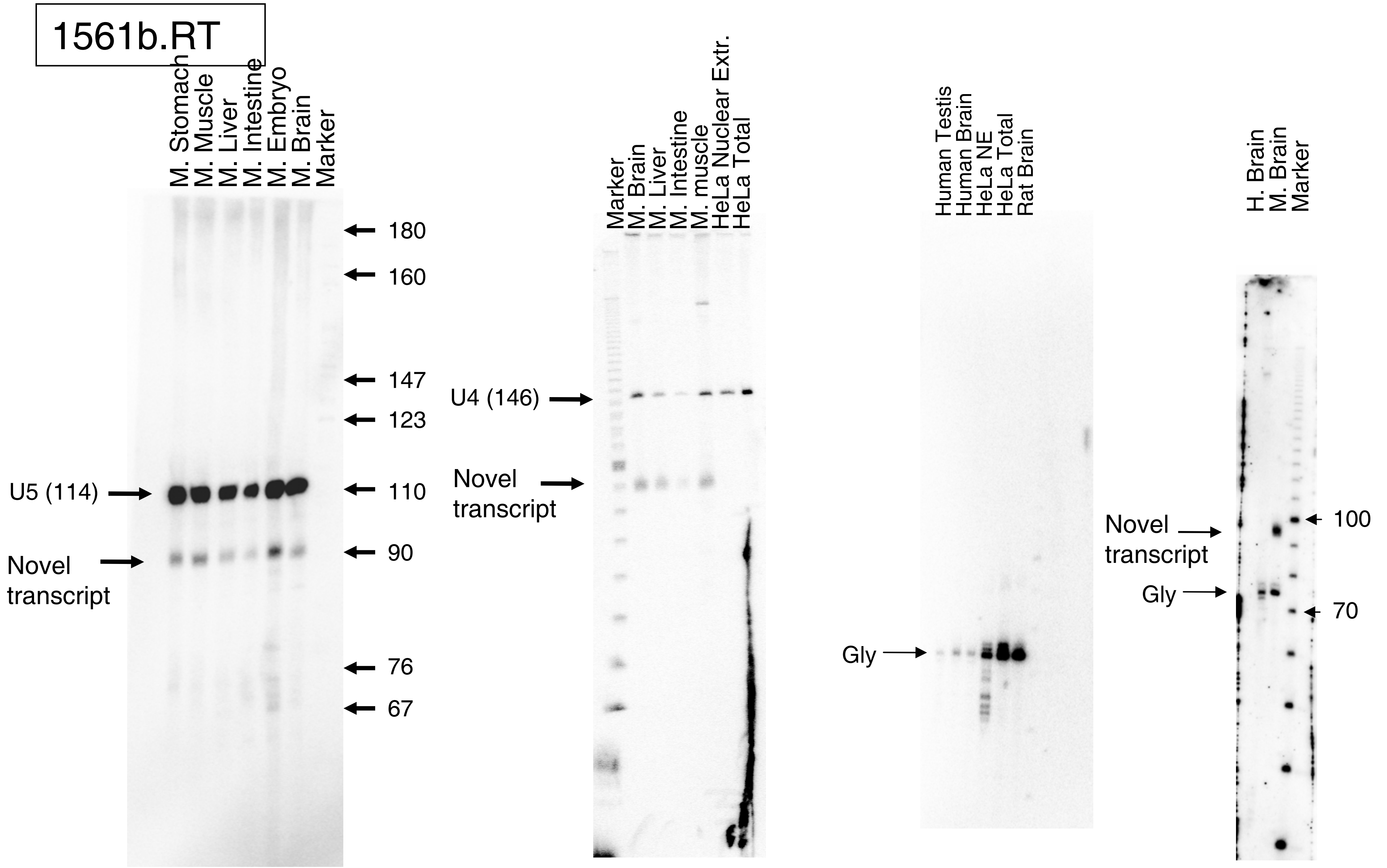

Hybridized with:	Mouse1561b.RT U5	Mouse 1561b.RT, human 1561b.RT U4	human 1561b.RT Gly-tRNA	Mouse 1561b.RT, human 1561b.RT Gly-tRNA

2921a

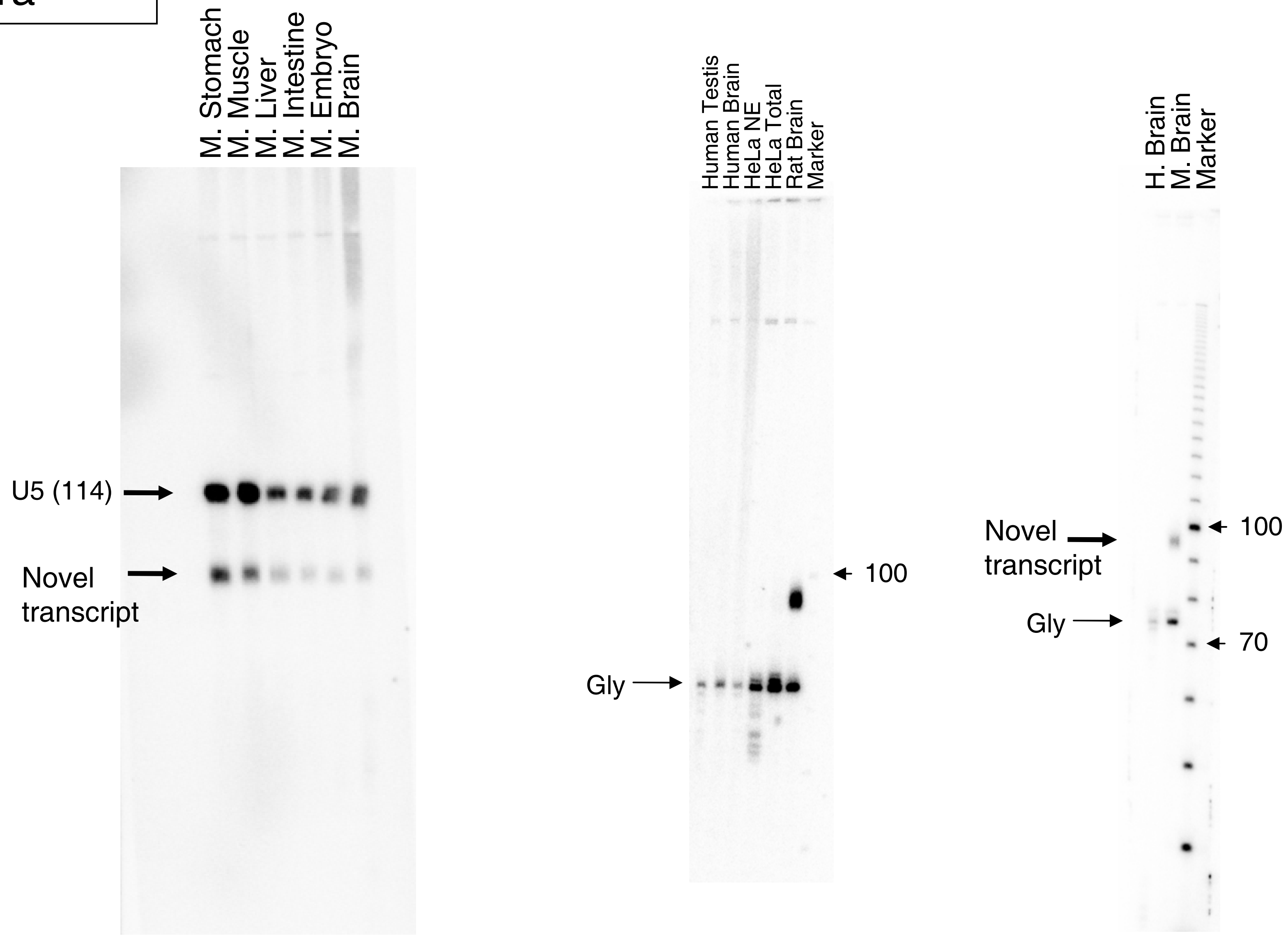

Hybridized with:	Mouse 2921a U5	human 2921a Gly-tRNA	Mouse 2921a, human 2921a, Gly-tRNA

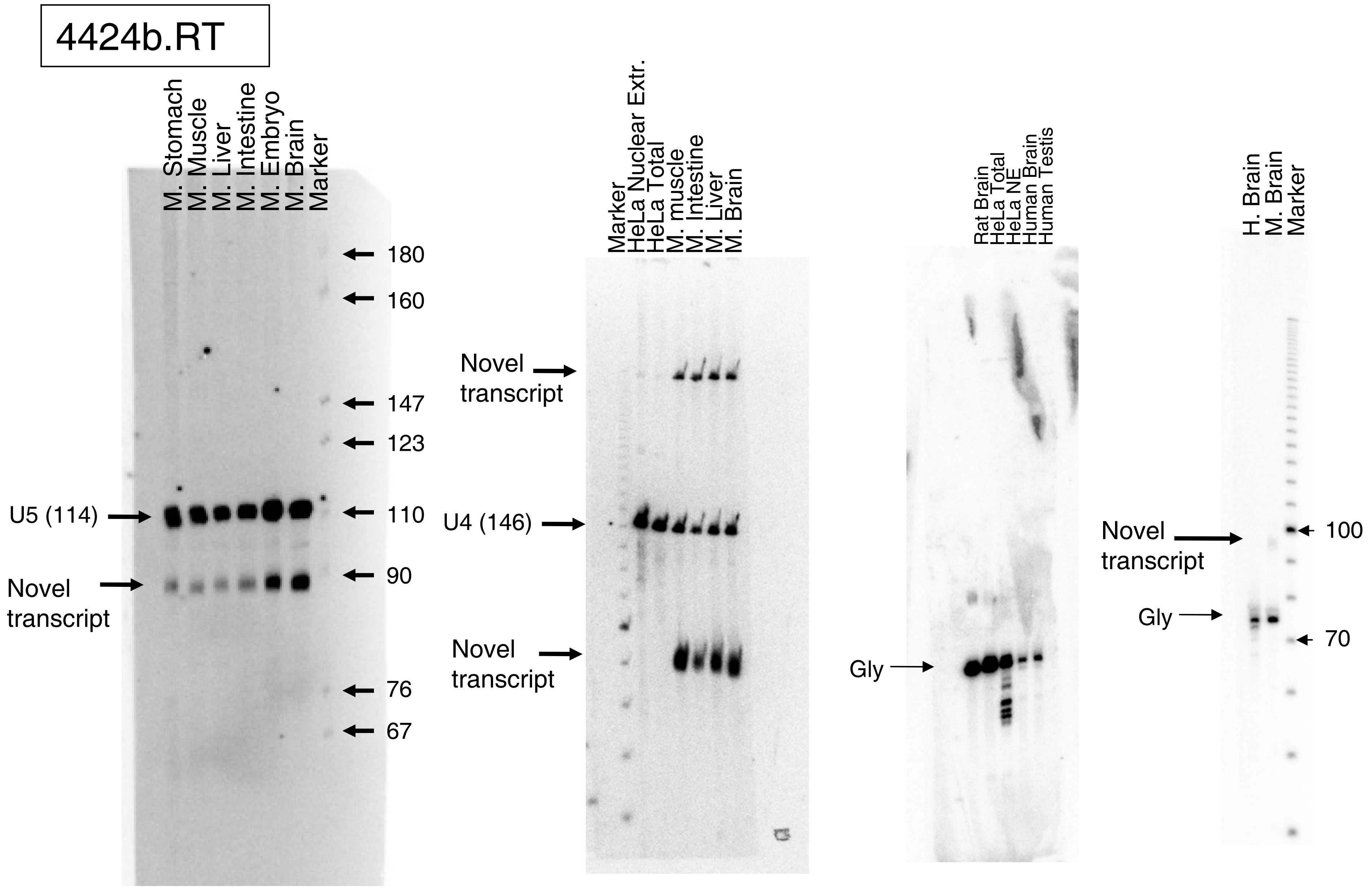

Hybridized with:	Mouse 4424b.RT<br>U5	Mouse 4424b.RT,<br>human 4424b.RT<br>U4	human 4424b.RT<br>Gly-tRNA	Mouse 4424b.RT,<br>human 4424b.RT<br>Gly-tRNA

2336c.RT

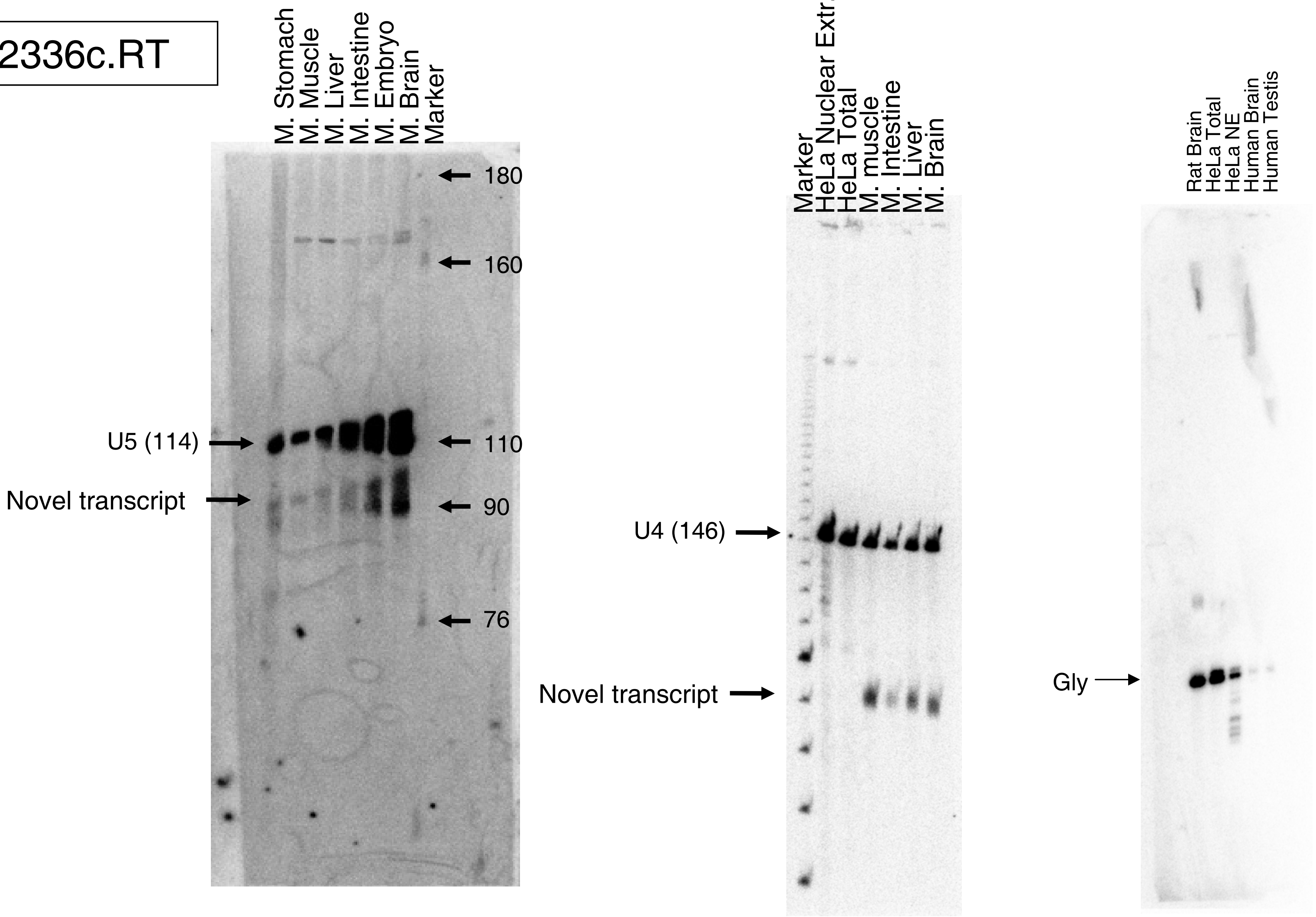

Hybridized with:	Mouse 2336c.RT U5	Mouse 2336c.RT, human 2336c.RT U4	human 2336c.RT Gly-tRNA

TB3942c

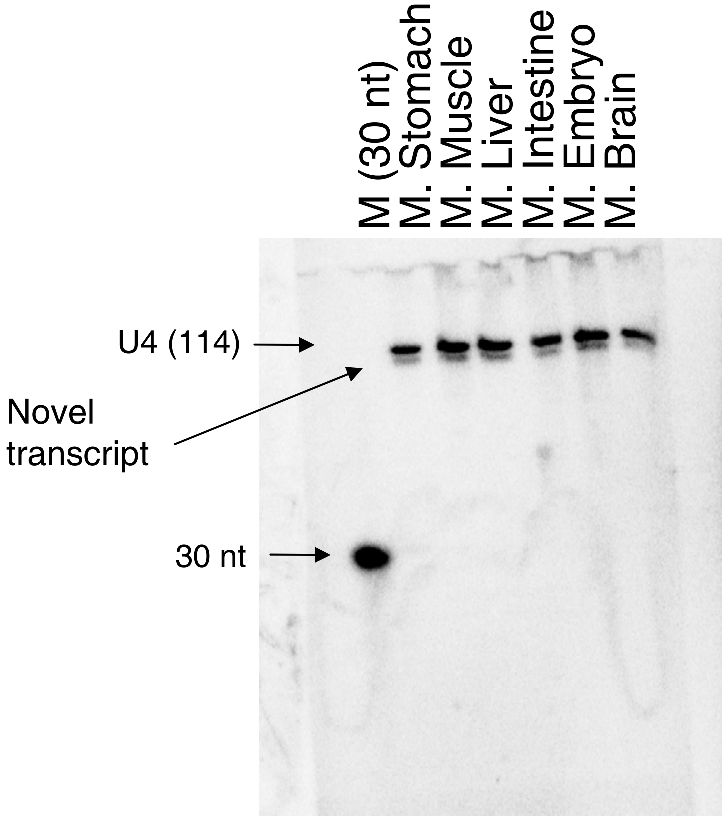

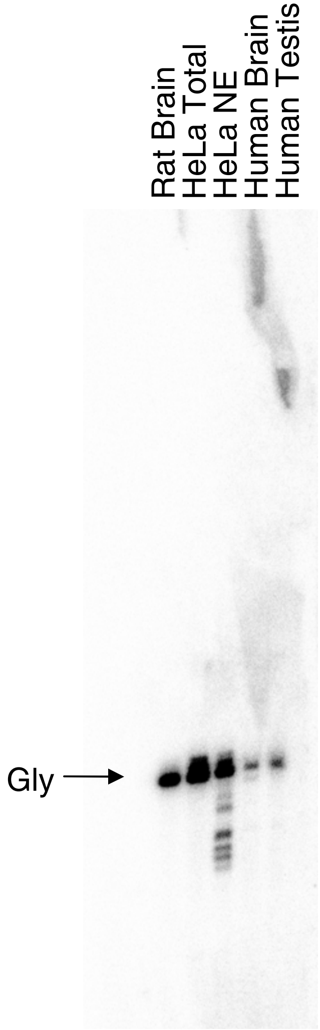

Hybridized with:

MouseTB3942c
U4

human TB3942c
Gly-tRNA

4299a

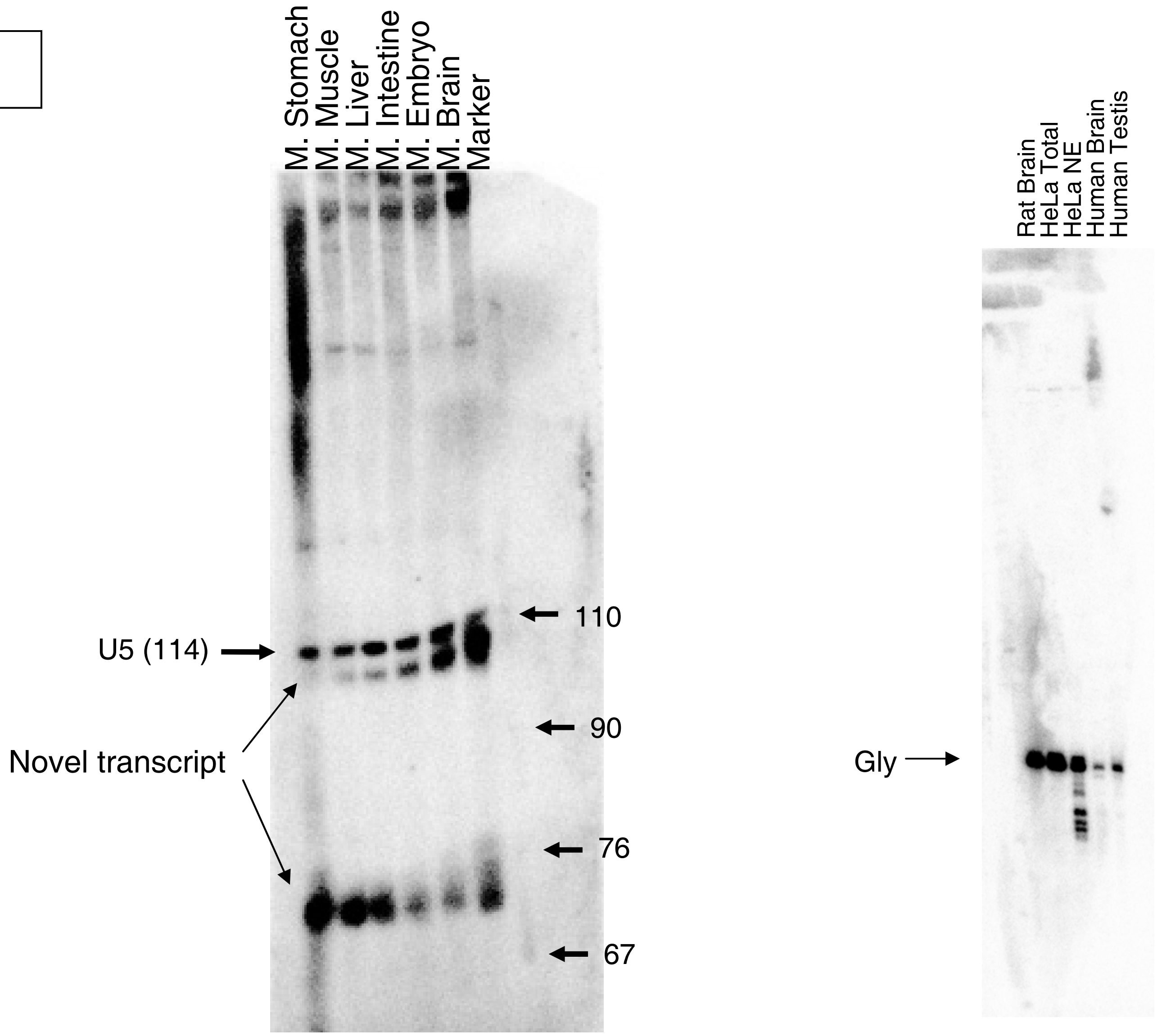

Hybridized with:	Mouse 4299a<br>U5	human 4299a<br>Gly-tRNA

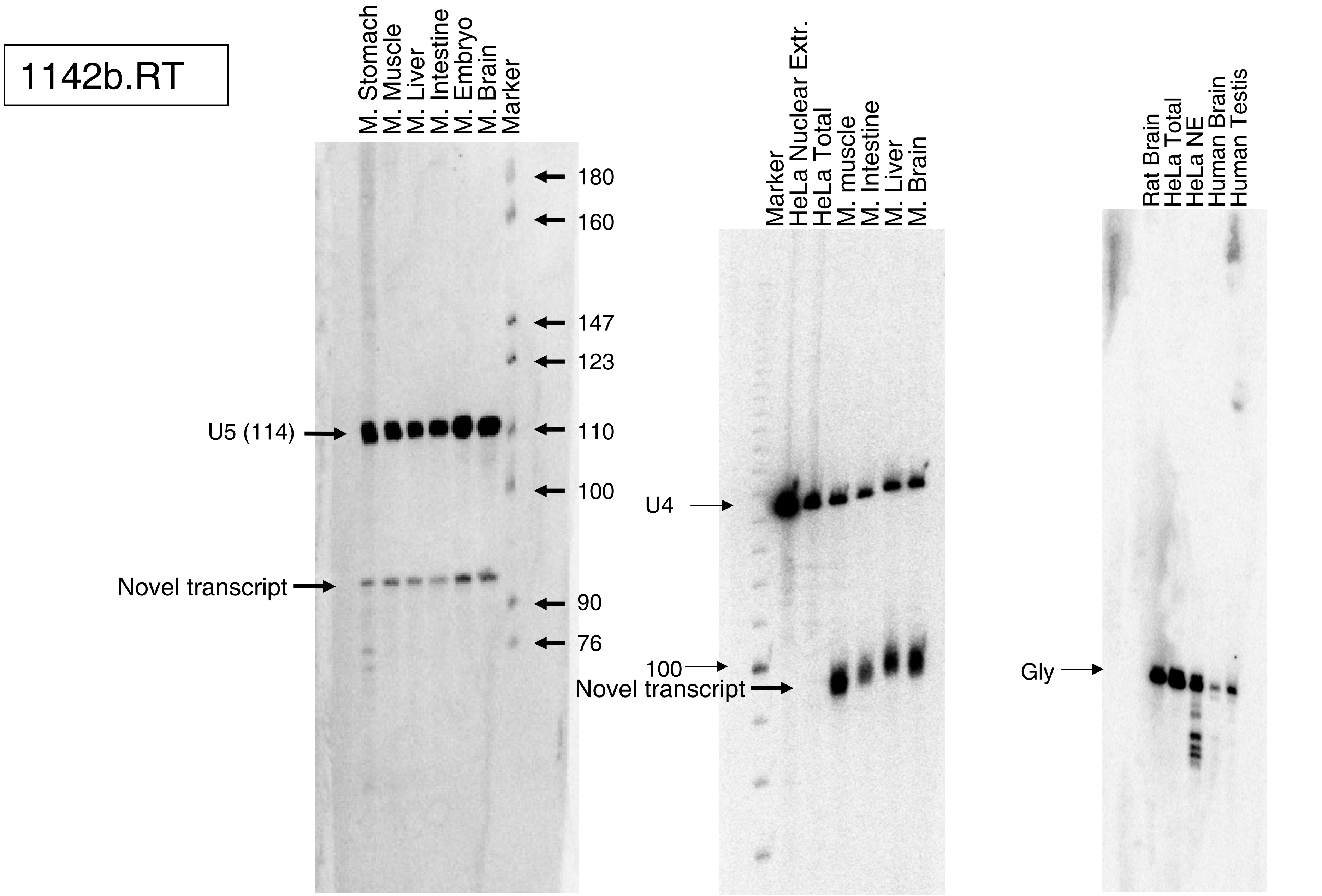

Hybridized with:	Mouse 1142b.RT<br>U5	Mouse 1142b.RT,<br>human 1142b.RT<br>U4	human 1142b.RT<br>Gly-tRNA
