# Supplementary material for: A systematic search for new mammalian noncoding RNAs indicates little conserved intergenic transcription
Source: BMC Genomics. 2005 Aug 5;6:104. doi: 10.1186/1471-2164-6-104 (PMC1199595; doi:10.1186/1471-2164-6-104)
Supplement: Additional File 5 — List of northern probe sequences. [file 1471-2164-6-104-S5.pdf]

Transcript ID	mouse probes	human probes
TB2921a	CATCTCTGTGGTCCTCCTGCCTTCT	CACATTCCTGTGGTCCTCCTGCCTTCT
TB1142b.RT	CAATAAATGGCTCCCGCGTGCTTCATTAA	CAATAAATGGCTCCCATGAGCTTCATTAA
TB4119a.RT	TCCTGGTGGTGTCCTGTAACCATGATAAC	TCCTGATGGTGTCGTGTAACCATGATAAC
TB4424b.RT	TCTGGAATCCATCCTTGCCTCTGTCC	TCTGGAATCCATCCTTGTCTCTGTCC
TB2336c.RT	CCTCCTGCTTTCCTGCCTTCTCAAC	CCTCCCGCCTTCCTGCCTCCTTAGC
TB4299a	GTTACCCTTCCCACCAGTCTCCCTAA	CTTACCCTTCCCACCAGTCTCCCTAA
TB337a.RT	GCCCTGCTCTCCCTTCAGTAAGTTTAC	GCCCTGCACTCCCTTCAGTAAG
TB3942c.RT	GTGAAGTGATGCATGCAAAGGAGGCAG	CTGCCTTCTTTACATGCATGGCTTCAC
TB1561b.RT	GCCCATCTGTCCTCTTACCTCCTTG	CCCCATCTGTCCTCTTACCTCCTTA

Positive controls	
U4	CCTCGGATAAACCTCATTGGCTA
U5	CTCTCCACGGAAATCTTTAGTAAAAGG
Gly-tRNA	GAATTCTACCACTGAACCACCAATGC
7SK	ACCTTGAGAGCTTGTTTGGAGGTTCTAGCAGGGGAGCGCAGCTACTCGTA
